# LncRNA PCGEM1 facilitates cervical cancer progression via miR-642a-5p/KIF5B axis

**DOI:** 10.32604/or.2024.047454

**Published:** 2024-06-20

**Authors:** YUANLIN LIU, YAN LIU, YAN WANG, QIANG WANG, YAN YAN, DANDAN ZHANG, HUIQIN LIU

**Affiliations:** 1 Department of Obstetrics and Gynecology, Shanghai First Maternity and Infant Hospital, School of Medicine, Tongji University, Shanghai, 200092, China; 2 Department of Obstetrics and Gynecology, The Second People’s Hospital of Nantong City, Nantong, 226002, China; 3 Department of Obstetrics and Gynecology, Affiliated Hospital of Nantong University, Nantong, 226002, China

**Keywords:** Cervical cancer, PCGEM1, KIF5B, MiR-642a-5p, tumorigenesis

## Abstract

At present, the role of many long non-coding RNAs (lncRNAs) as tumor suppressors in the formation and development of cervical cancer (CC) has been studied. However, lncRNA prostate cancer gene expression marker 1 (PCGEM1), whose high expression not only aggravates ovarian cancer but also can induce tumorigenesis and endometrial cancer progression, has not been studied in CC. The objective of this study was to investigate the expression and the underlying role of PCGEM1 in CC. The relative expression of PCGEM1 in CC cells was detected by real-time PCR. After the suppression of PCGEM1 expression by shRNA, the changes in the proliferation, migration, and invasion capacities were detected via CCK-8 assay, EdU assay, and colony formation assay wound healing assay. Transwell assay and the changes in expressions of epithelial-to-mesenchymal transition (EMT) markers were determined by western blot and immunofluorescence. The interplay among PCGEM1, miR-642a-5p, and kinesin family member 5B (KIF5B) was confirmed by bioinformatics analyses and luciferase reporter assay. Results showed that PCGEM1 expressions were up-regulated within CC cells. Cell viabilities, migration, and invasion were remarkably reduced after the suppression of PCGEM1 expression by shRNA in Hela and SiHa cells. N-cadherin was silenced, but E-cadherin expression was elevated by sh-PCGEM1. Moreover, by sponging miR-642a-5p in CC, PCGEM1 was verified as a competitive endogenous RNA (ceRNA) that modulates KIF5B levels. MiR-642a-5p down-regulation partially rescued sh-PCGEM1’s inhibitory effects on cell proliferation, migration, invasion, and EMT process. In conclusion, the PCGEM1/miR-642a-5p/KIF5B signaling axis might be a novel therapeutic target in CC. This study provides a research basis and new direction for targeted therapy of CC.

## Introduction

Cervical cancer (CC) ranks 2nd in the global cancer incidence and 4th in terms of worldwide mortality [1]. Traditional treatment options for CC include radical hysterectomy and chemoradiotherapy [2]. Nevertheless, in clinical settings, the majority of patients with advanced or metastatic CC have a poor prognosis [3]. Consequently, it is crucial to learn about the therapeutic targets in the progression of CC.

Long non-coding RNAs (lncRNAs), as a subclass of non-coding RNA with a length more than 200 nucleotides (nt), are located in the nucleus and cytoplasm and exert vital effects on both physiological and pathological processes by controlling the expression of genes involved in those processes [4]. At present, the role of lncRNAs as tumor suppressors in the formation and development of malignant tumors including CC has been studied. For instance, the knockdown of small nucleolar RNA host gene 14 (SNHG14) inhibited tumor cell proliferation and promoted apoptosis [5]. Higher LINC00511 expression predicted a poor prognosis of CC, and silencing LINC00511 expression suppressed CC cell proliferation, metastasis, and invasion [6]. Ectopic GAS5 could attenuate CC cell proliferation and motility [7].

A new long noncoding RNA (lncRNA), prostate cancer gene expression marker 1 (PCGEM1), is located on chromosome 2q32.3, which is overexpressed in prostate cancer [8,9]. The oncogene PCGEM1 has been reported to be activated in a variety of malignancies. For example, Chen et al. indicated that up-regulated PCGEM1 deteriorated the malignant behaviors of ovarian cancer by interacting with RhoA [10]. Previous study has shown that PCGEM1 over-expression induced tumorigenesis and endometrial carcinoma progression by regulating miR-129-5p/STAT3 signaling [11]. Zhang et al. found that silencing of PCGEM1 expression memorably modulated EMT to further descend gastric cancer metastasis [12]. Although PCGEM1 has been found to have carcinogenic effects in various malignancies, its possible role in the development of CC was unknown. This study concentrated on examining the roles played by PCGEM1 in CC as well as the underlying mechanism.

## Materials and Methods

### Cell lines

DMEM/F12 (Thermo Fisher Scientific, USA) containing 10% FBS (Gibico, NY, USA) was used to cultivate MAC-T, which was supplied by ATCC (USA). The cells were incubated in the humid incubator at 37°C and 5% CO_2_ during incubation. Ect1/E6E7 and CC cells (HeLa, CaSki, and SiHa) were supplied by ATCC (USA) and cultivated in DMEM media with 10% FBS (Gibico, NY, USA), which was kept in a humid incubator at 37°C and 5% CO_2_.

### Cell transfection

CC cell lines were cultured in six-well plates except for cell counting in 24-well plates. The cationic liposome transfection method was used according to the manufacturer’s protocol. In brief, the fresh antibiotics-free cell culture medium(Thermo Fisher Scientific, USA) was changed, and inoculated 1.0 × 10^6^ cells into each well the day before transfection. Cells were treated with shRNA in the presence of Lipofectamine2000 (Invitrogen) and the ratio of vector/Lipofectamine2000 was 1:3, incubated at serum-free and non-antibiotics media for 6–8 h, then changed to the full media with 10% FCS till 48 h. Short hairpin RNA (shRNA)-expressing lentivirus vectors were created by Genechem to target PCGEM1, shRNA negative controls (sh-NC), NC mimic/miR-642a-5p mimic, and NC inhibitor/miR-642a-5p inhibitor (Ribobio, Shanghai, China). Each experiment was performed in triplicate and the standard deviation was obtained.

### Real-time PCR

Total RNA was isolated from CC cell lines using TRIzol reagent (Invitrogen) according to the manufacturer’s instructions. cDNA synthesis for coding genes was performed with 1 μg of total RNA according to the manufacturer’s instructions (Takara, Tokyo, Japan). The mRNA levels of PCGEM1, KIF5B, and miR-642a-5p were analyzed using SYBR PCR Master Mix reagent kits (Takara, Tokyo, Japan) according to the manufacturer’s instructions. GAPDH was amplified as a control. The relative expression levels of CCKBR were calculated using the 2^−ΔΔCT^ method. The specific primers as shown in Table 1.

**Table 1:** Primer sequences

Gene name	Primer sequences
PCGEM1	F: 5′-tccacccaatacacaggat--3′
	R: 5′-aattgggagctgatgaggac-3′
KIF5B	F: 5′-AGACAAAAACCGAGTTCCCTATG-3′
	R: 5′-CGATCACGACCGTGTCTTCT-3′
GAPDH	F: 5′-TGTGGGCATCAATGGATTTGG-3′
	R: 5′-ACACCATGTATTCCGGGTCAAT-3′
miR-642a-5p	F: 5′-GCGGTCCCTCTCCAAATGT-3′
	R: 5′-AGTGCAGGGTCCGAGGTATT-3′
GAPDH	F: 5′-aaactggaacggtgaaggtg-3′
	R: 5′’-agagaagtggggtggctttt-3′

### CCK-8 assay

Cell viability was evaluated by a CCK-8 (Beyotime Biotechnology, Shanghai, China) according to the manufacturer’s instructions. Different groups’ transfected CC cells (2 × 10^3^) were seeded into 96-well plates. CCK-8 kit (Sigma, USA) was utilized after being incubated for 0 and 48 h. Using a spectrophotometer (Molecular Devices, San Jose, USA), absorbance (OD) (450 nm) was detected.

### EdU assay

Transfected CC cells from various groups were given a 30-minute incubation period with the EdU reaction solution. CC cells were then trypsinized, fixed with formaldehyde, and washed in PBS containing 1% BSA. PBS was used to rinse the cells after Triton X-100 permeabilization and fluorescence microscopy was used to see the cells.

### Colony formation assay

In brief, CC cells were inoculated into six-well plates. After 48 h of transfection, CC cells were cultured in a fresh medium and the medium should be updated every 3 days during culture. After 14 days, CC cells were gently washed with PBS, followed by fixing with paraformaldehyde (4%, Beyotime) for 0.5 h at 4°C. Then, these cells were again washed with PBS, followed by staining with crystal violet (0.1%, Beyotime) for 2 h. A microscope (Olympus, Tokyo, Japan) was employed for counting the colonies (>50 cells per colony).

### Wound-healing assay

The proper density of transfected CC cells (5 × 10^5^/well) was added to 6-well plates. A wound was scraped once the cells had 80% confluence. A light microscope (Nikon, Japan) was used to take photos of cells pictures at 0 and 48 h (200×).

### Transwell assay

For invasion experiments, Matrigel was evenly spread on the Transwell chamber and dissolved in serum-free DMEM for one hour at 37°C. CC cells from the various groups (sh-NC, sh-PCGEM1, sh-PCGEM1+miR-642a-5p NC, sh-PCGEM1+miR-642a-5p inhibitor) were planted into the top chambers at the right density (10^5^/well) for the migration and invasion experiments. Into the bottom chambers was put a medium containing 10% FBS. Migrated and invaded cells were stained with crystal violet (0.1%) and photographed using a light microscope (Nikon, Japan) after 48 h of incubation at 37°C at room temperature (200×).

### Western blot

Protein was extracted from CC cell lines using RIPA buffer (50 mM Tris (pH 7.4), 150 mM NaCl, 1% v/v Triton X-100, 1% v/v sodium deoxycholate, 0.1% v/v SDS containing protease and phosphatase inhibitors (100 mmol/L PMSF, 1 mg/mL leupeptin, 1 mg/mL aprotinin)) (Beyotime, China). Total proteins (60 µg) were resolved by SDS-PAGE and transferred to a polyvinylidene fluoride membrane (PVDF, Bio-Rad, Hercules, CA, USA). The polyvinylidene fluoride membranes were incubated with the primary and secondary antibodies. Membranes were then incubated with primary antibodies overnight at 4°C, using 5% skimmed milk. Anti-KIF5B (1:2,000, bs-24715R, Bioss) and anti-GAPDH (1:2,000, bs-0755R, Bioss) antibodies are used, with GAPDH serving as the endogenous control. Next, membranes were treated with a secondary antibody for an additional hour at 37°C (1:2, 000, bs-0311P-HRP, Bioss). Finally, protein blots were examined using ECL (Millipore, USA). Densitometry was performed using image-J software (1.45 s, National Institutes of Health, USA).

### Immunofluorescence

The CC cell slices from different groups were sealed for 30 min with 3% bovine serum protein (Gibico, NY, USA) at 37°C. Then slides were incubated overnight with primary antibodies at 4°C. Then slices were incubated with second antibody and DAPI for 60 and 15 min, respectively, at room temperature with DAPI. Fields were photographed in each slice using a microscope (Leica Microsystems, Wetzlar, Germany).

### luciferase reporter assay

In this study, PCGEM1 or KIF5B WT/MUT were sub-cloned to produce pmirGLO-PCGEM1 or pmirGLO-KIF5B WT/MUT, which were then co-transfected into CC cells along with NC mimics or miR-642a-5p mimics. The luciferase activity was assessed after co-transfection for 48 h (Promega, USA).

### Statistical analysis

Data from three repeats are shown by the mean ± standard deviation (SD). The Software used is GraphPad Prism 5.0 (GraphPad Software, Inc.). The normal distribution was used S-W test. The difference with *p* < 0.05 was considered statistically significant, according to the *t*-test used to compare the two groups, the single-factor ANOVA used to compare the groups, and the Tukey post-test. Six parallels were set for each treatment group *in vitro* experiments, and quantification of microscopy results was performed by randomly selecting 10 representative fields from each group for quantitative analysis.

## Results

### PCGEM1 knockdown suppressed CC cell proliferation

**Figure 1:**
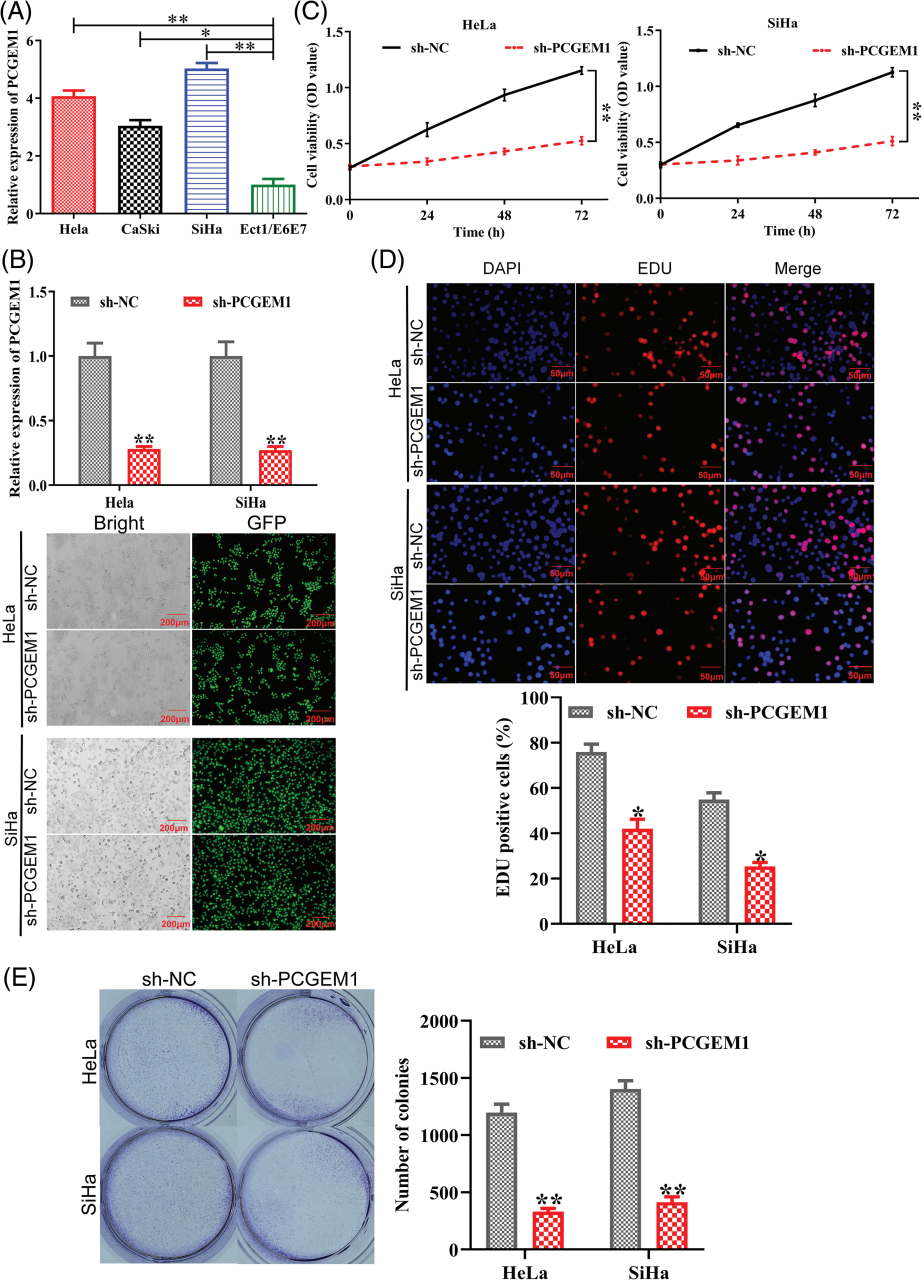
PCGEM1 knockdown suppressed CC cell proliferation. (A) Three human CC cell lines (HeLa, CaSki, and SiHa) and healthy human cervical epithelial Ect1/E6E7 cells were used in the real-time PCR analysis of PCGEM1 expression. *p < 0.05, **p < 0.01 *vs*. Ect1/E6E7 cells. (B) Sh-PCGEM1 or sh-NC were transfected into Hela and SiHa cells, respectively. Real-time PCR and the evaluation of GFP expression provided evidence of the transfection’s effectiveness (scale bar, 25 µm). (C) CCK-8, (D) EdU (scale bar, 50 µm), and (E) formation of colonies assays were adopted to ascertain the proliferation of sh-PCGEM1-transfected Hela and SiHa cells. *p < 0.05, **p < 0.01 *vs*. sh-NC group.

Herein, RT-PCR was employed for assessing PCGEM1 expressions within CC cells and Ect1/E6E7 cells. In CC cells, PCGEM1 expression was up-regulated, especially in Hela, SiHa and CaSki cells, as shown in Fig. 1A. As demonstrated by RT-PCR and GFP fluorescence microscopy, PCGEM1 expressions within Hela and SiHa cells was silenced by shRNA transfection (Fig. 1B). CCK-8, EdU and formation of colonies were conducted for determining sh-PCGEM1’s effects on CC cell proliferation. As shown in (Fig. 1C), the Cell viability of Hela and SiHa cells was remarkably reduced after the suppression of PCGEM1 expression by shRNA. From the results of EdU assays, the proportion of EdU-positive cells was reduced after the suppression of PCGEM1 expression by shRNA (Fig. 1D). Additionally, the knockdown of PCGEM1 significantly decreased colony formation ability in Hela and SiHa cells (Fig. 1E).

### PCGEM1 depletion reduced migration and invasion of CC cells

**Figure 2:**
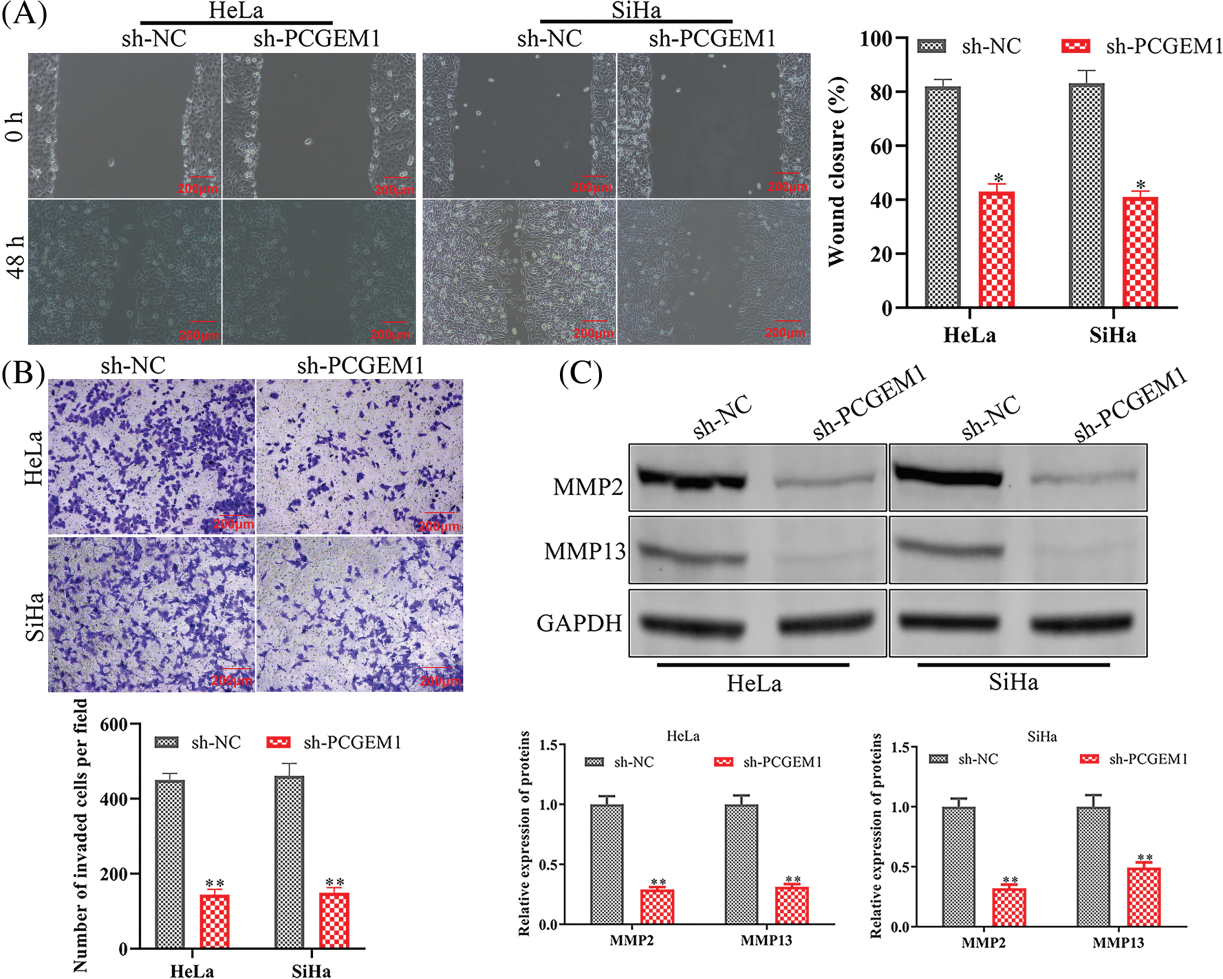
PCGEM1 depletion reduced migration and invasion of CC cells. (A and B) The ability of sh-PCGEM1-transfected Hela and SiHa cells to migrate and invade was evaluated using the wound-healing assay (scale bar, 100 µm) and the Transwell assay (scale bar, 50 µm). (C) The expression of MMP-2 and MMP-13 in sh-PCGEM1-transfected Hela and SiHa cells was determined using the western blot assay. *p < 0.05, **p < 0.01 *vs*. sh-NC group.

To explore PCGEM1’s impacts on CC cell metastasis, wound healing, and Transwell, as well as western blotting analyses of associated proteins were conducted when PCGEM1 was down-regulated. The functional analysis demonstrated that PCGEM1 down-regulation reduced Hela and SiHa cell migration (Fig. 2A) and invasion (Fig. 2B) capacities by decreasing matrix metalloproteinases (MMP-2, and MMP -13) (Fig. 2C).

### PCGEM1 inhibition repressed the EMT program

**Figure 3:**
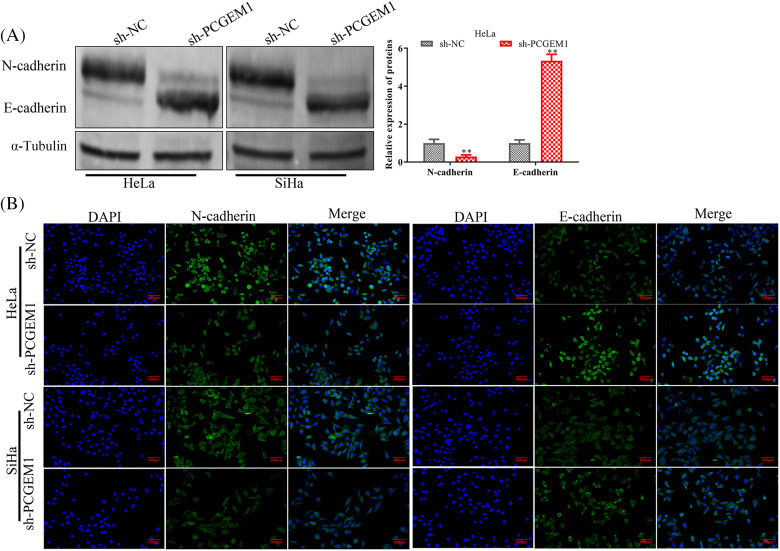
PCGEM1 inhibition repressed the EMT program. In Hela and SiHa cells transfected with sh-PCGEM1, the expression levels of N- and E-cadherin were measured using Western blot assay (A) and immunofluorescence (B) assays (scale bar, 50 µm). **p < 0.01 *vs*. sh-NC group.

Whether PCGEM1 affected EMT progression in CC cells was further investigated. The western blot results indicated PCGEM1 silencing significantly down-regulated the mesenchymal marker N-cadherin, along with increasing E-cadherin expressions, an epithelial marker (Fig. 3A). Similarly, results of the immunofluorescence analysis revealed that N-cadherin was silenced, but E-cadherin expression was elevated by sh-PCGEM1 (Fig. 3B).

### PCGEM1 regulated KIF5B by competing for miR-642a-5p

**Figure 4:**
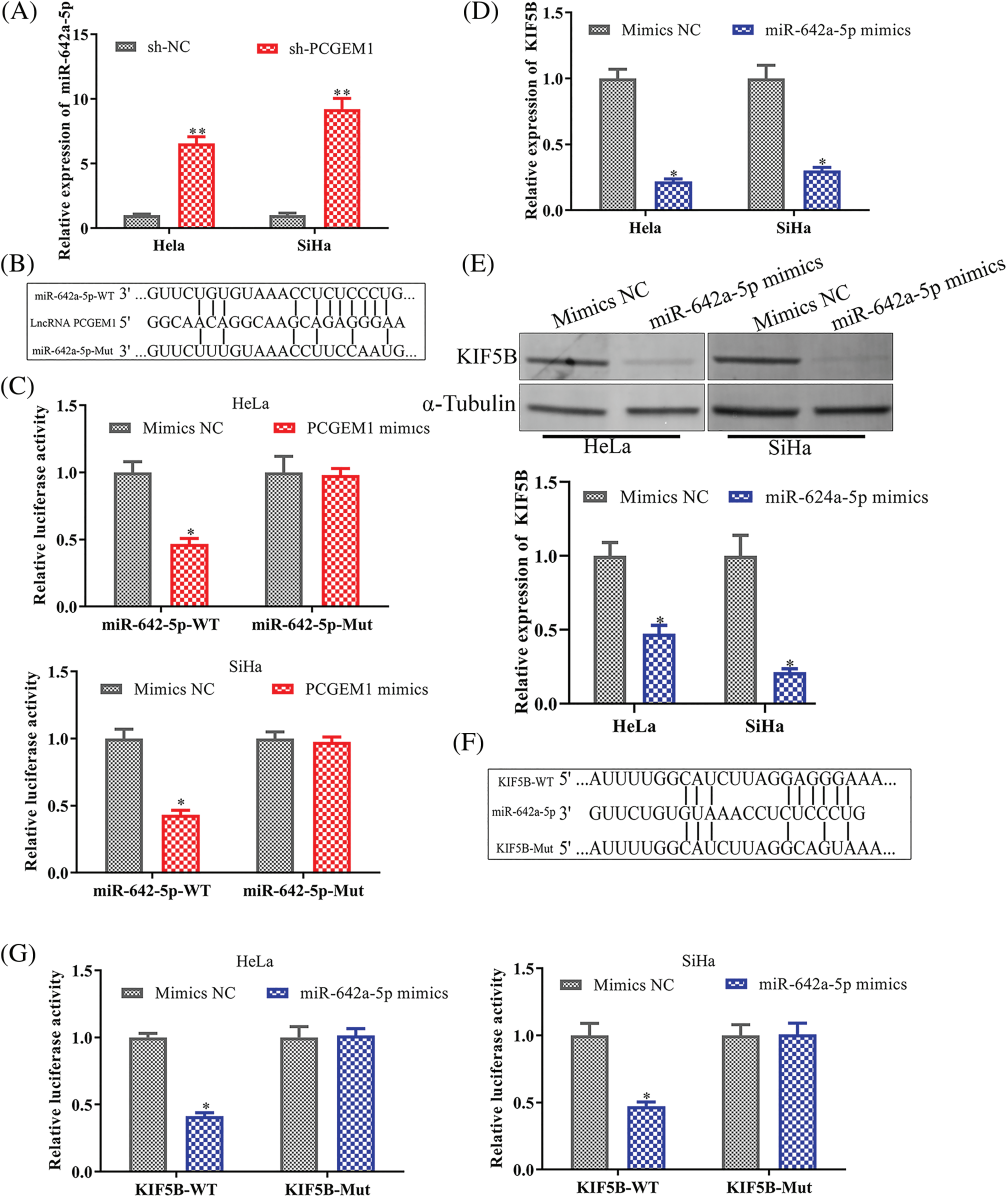
PCGEM1 regulated KIF5B expression by competing for miR-642a-5p. (A) The mRNA expression of miR-642a-5p was measured in Hela and SiHa cells transfected with sh-PCGEM1 or sh-NC. **p < 0.01 *vs*. sh-NC group. (B) The conceptual representation of the miR-642a-5p combining sites in PCGEM1. (C) In Hela and SiHa cells, the luciferase reporters harboring WT or MUT miR-642a-5p were co-transfected with PCGEM1 mimic or mimic NC. *p < 0.05 *vs*. Mimics NC group. (D–E) In Hela and SiHa cells transfected with miR-642a-5p mimic or mimic NC, the mRNA and protein levels of KIF5B were assessed. *p < 0.05 *vs*. Mimics NC group. (F) The miR-642a-5p putative targeting sites in the WT and MUT of KIF5B are shown schematically. (G) In Hela and SiHa cells, the luciferase reporters harboring WT or MUT KIF5B were co-transfected with miR-642a-5p mimic or mimic NC. *p < 0.05 *vs*. Mimics NC group.

LncRNAs could serve as miRNA sponges, thereby modulating mRNA expression [13]. We investigated whether PCGEM1-mediated tumorigenesis might operate through a competitive endogenous RNA (ceRNA) regulatory mechanism. PCGEM1 inhibition could significantly enhance miR-642a-5p levels within CC cells (Fig. 4A). MiR-642a-5p combining sites in PCGEM1 were displayed in Fig. 4B. The PCGEM1 mimic lowered the luciferase activity of the miR-642a-5p WT reporter vector, but not the mutant reporter, according to a dual-luciferase experiment (Fig. 4C). MiR-642a-5p over-expression could diminish KIF5B levels, an oncogene within CC cells (Figs. 4D and 4E). The 3′UTR region of KIF5B was predicted by a bioinformatics study to include numerous combining sites for miR-642a-5p (http://www.targetscan.org/) (Fig. 4F). Based on Fig. 4G, the activities of WT-KIF5B 3'-UTR luciferase were observably decreased by miR-642a-5p mimic (Fig. 4G). Collectively, these findings revealed that PCGEM1 competed with KIF5B for miR-642a-5p binding in CC cells.

### PCGEM1 silencing inhibited proliferation, metastasis, invasion and EMT in CC cells by regulating the miR-642a-5p/KIF5B axis

**Figure 5:**
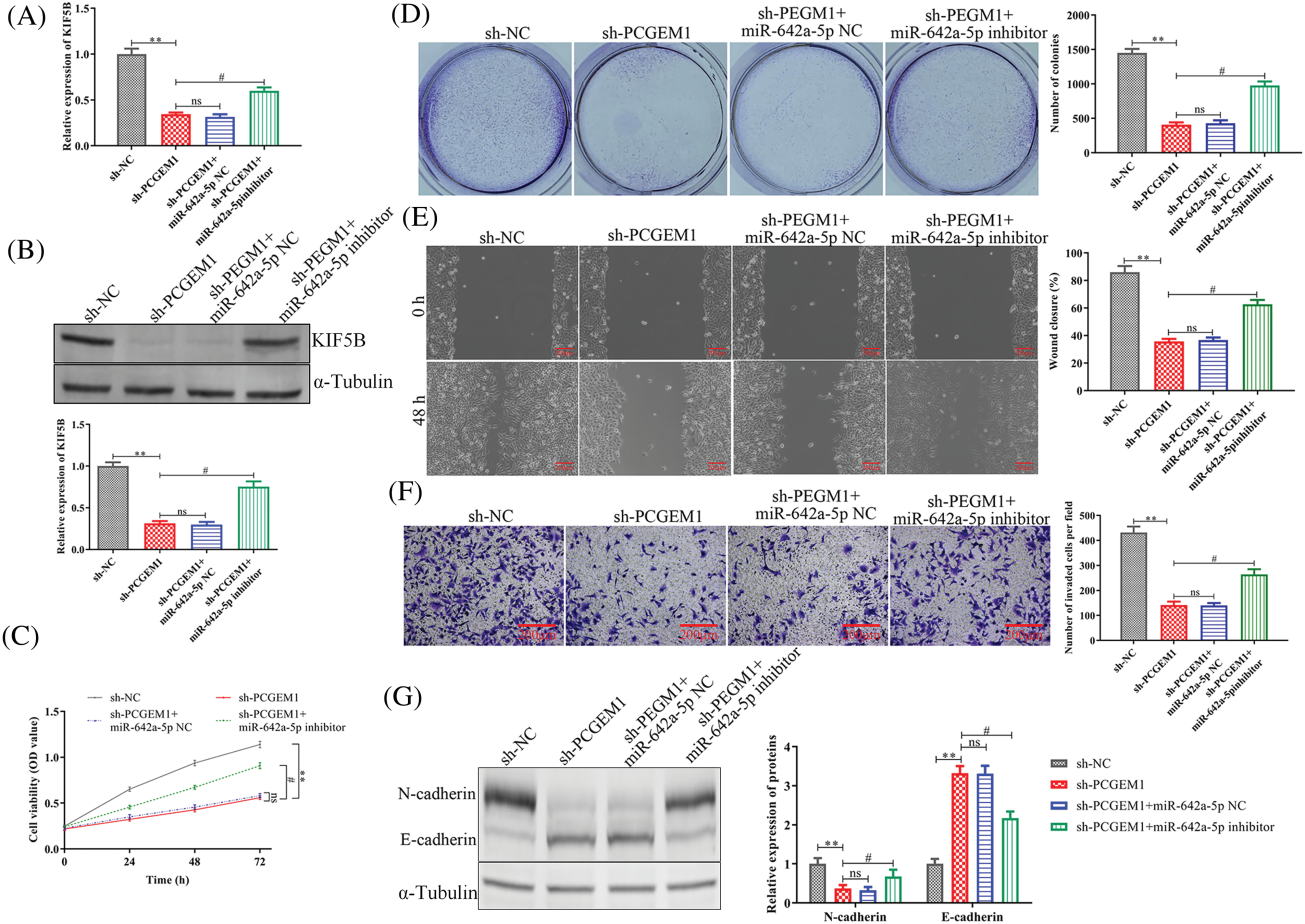
PCGEM1 silencing inhibited proliferation, metastasis, invasion, and EMT in CC cells by the regulation of miR-642a-5p/KIF5B axis. (A and B) The mRNA and protein levels of KIF5B, (C) CCK-8 assay and colony formation assay, (D) formation of colonies assays, (E) wound-healing assay (scale bar, 100 µm), (F) Transwell assay (scale bar, 50 µm), and (G) the protein levels of N-cadherin and E-cadherin were determined in SiHa cells transfected with sh-NC or sh-PCGEM1 alone, or together with miR-642a-5p inhibitor or inhibitor NC. **p < 0.01 *vs*. sh-NC group, ^#^*p* < 0.01 *vs*. sh-PCGEM1+miR-642a-5p NC group.

Finally, we investigated whether miR-642a-5p inhibitor could antagonize the anti-tumor effects of sh-PCGEM1. As expected, KIF5B levels were suppressed by sh-PCGEM1, which was attenuated by transfection with a miR-642a-5p inhibitor within SiHa cells (Figs. 5A and 5B). Further investigation illustrated that miR-642a-5p down-regulation partially rescued sh-PCGEM1’s inhibitory effects on cell proliferation (Figs. 5C–5D), migration (Fig. 5E), invasion (Fig. 5F) and EMT process (Fig. 5G). Taken together, these results demonstrated that PCGEM1 exerted carcinogenic effects in CC cells via the miR-642a-5p/KIF5B axis.

## Discussion

As a carcinogenic lncRNA, PCGEM1 contributed to the occurrence and development of prostate cancer [14,15], ovarian cancer [10], endometrial carcinoma [11], and gastric cancer [12,16]. However, there was few report about the biological roles of PCGEM1 in CC. In this study, we for the first time found CC cells contain increased PCGEM1 levels. The majority of deaths connected to cancer are still caused by metastasis. In this research, we investigated the PCGEM1 functions on PCGEM1 in CC progression and revealed PCGEM1 down-regulation inhibited CC cell proliferation, metastasis, and invasion *in vitro*. EMT, an essential progress in CC, mediated by lncRNAs has been implicated in diverse tumors. For instance, SNHG7 was described as an EMT-promoter in breast cancer cells, where it sponged miR-34a, leading to the activation of the Notch-1 pathway [17]. H19 promoted esophageal cancer cell metastasis via negatively regulating let-7c, an anti-EMT miRNA [18]. Fer-1-like protein 4 (FER1L4) prevented EMT initiation, controlling osteosarcoma cell proliferation via miR-18a-5p/SOCS5/PI3K/AKT signaling pathway [19]. As indicated by western blot and immunofluorescence analysis, the knockdown of PCGEM1 expression dramatically inhibited the EMT process by down-regulating N-cadherin but up-regulating E-cadherin within CC cells.

Although increased PCGEM1 expression has been found in CC cells, the detailed regulatory mechanism by which PCGEM1 mediated the development of CC remains largely unclear. It has been demonstrated that lncRNAs act as post-transcriptional miRNA sponges. There is mounting evidence that lncRNAs act as post-transcriptional miRNA sponges in a new regulation mechanism. A previous study demonstrated that SNHG20 functioned as a ceRNA to target ZEB2 and RUNX2 by sponging miR-154 [20]. SNHG6 may contribute to breast cancer aggressiveness via the miR-26a/VASP axis [21]. Urothelial Cancer Associated 1 (UCA1) acted as ceRNA directly combining miR-498, a tumor suppressor, inhibiting its function in esophageal cancer cells [22]. Using bioinformatics databases (DIANA tools), PCGEM1 and KIF5B were predicted to share the regulatory sites for miR-642a-5p. Besides, further evidence was provided to support the regulatory connection between PCGEM1 and miR-642a-5p. (1) PCGEM1 inhibition increased miR-642a-5p levels. (2) Luciferase activity assay proved the target relationship between PCGEM1 and miR-642a-5p. Besides, miR-642a-5p could directly target KIF5B, a positive regulator of EMT [23,24]. Similarly, miR-642a-5p acted as a tumor suppressor in multiple tumor types, including Hodgkin lymphoma [25] and colon cancer [26]. These data provided evidence that PCGEM1 served as a ceRNA for miR-642a-5p to modulate KIF5B within CC cells. Furthermore, miR-642a-5p down-regulation restored the inhibition of CC cell proliferation, invasion, and EMT caused by PCGEM1 silencing.

Increasing evidence has shown that circular RNAs (circRNAs) can be used as valuable biomarkers for early detection of CC and have the potential to be a therapeutic target for intervention. The role of circRNAs in a variety of diseases such as cancer has been revealed. Not only as miRNA sponge, it affects the transport of intracellular RNA binding proteins (RBPs), regulates the expression of parental genes, and regulates protein translation, but also circRNAs have Janus-faced characteristics in cellular processes [27]. Whether PCGEM1 has a similar mechanism in CC requires further investigation.

Studies have shown that TUG1 [28], SNHG5 [29], MBNL1-AS1 [30], HOTAIR [31] and other lncRNAs are significantly increased in cervical cancer tissues and cell,associated with FIGO stage, lymph node metastasis, tumor size and differentiation in patients with cervical cancer. By regulating the expression of key genes, they regulate proliferation, apoptosis, cell cycle, migration and invasion, EMT and cancer stem cells, and participate in chemotherapy resistance, radiation resistance and immunotherapy. It is involved in the occurrence and development of cervical cancer [32]. Whether PCGEM1 has a similar mechanism in CC requires further investigation.

To sum up, PCGEM1 expressions were up-regulated within CC cells. Interference of PCGEM1 could inhibit CC cell proliferation and metastasis capacities, and EMT progression by acting as a ceRNA to modulate KIF5B levels through sponging miR-642a-5p, offering a novel perspective on the prevention and treatment of CC.

## Data Availability

All data generated or analyzed during this study are included in this published article.
