# Correction: LncRNA PCGEM1 facilitates cervical cancer progression via miR-642a-5p/KIF5B axis

**DOI:** 10.32604/or.2024.056768

**Published:** 2025-05-29

**Authors:** YUANLIN LIU, YAN LIU, YAN WANG, QIANG WANG, YAN YAN, DANDAN ZHANG, HUIQIN LIU

**Affiliations:** 1 Department of Obstetrics and Gynecology, Shanghai First Maternity and Infant Hospital, School of Medicine, Tongji University, Shanghai, 200092, China; 2 Department of Obstetrics and Gynecology, The Second People’s Hospital of Nantong City, Nantong, 226002, China; 3 Department of Obstetrics and Gynecology, Affiliated Hospital of Nantong University, Nantong, 226002, China

In the article “LncRNA PCGEM1 facilitates cervical cancer progression via miR-642a-5p/KIF5B axis” (*Oncology Research*, 2024, Vol. 32, No. 7, pp. 1221–1229. doi: 10.32604/or.2024.047454), there were some errors in the content. In order to ensure the scientific and rigorous nature of our academic publications, we deleted the incorrect content that is not related to this study, supplemented the details of the method, and replaced the retracted reference. The corrections do not change any results or conclusion of the article. We apologize for any inconvenience caused.

The authors would like to correct the content below:

**Table:** 

Page. No.	Exact content to be corrected	Correction
1222	Delete the MAC-T cell lines in the section **“Materials and Methods-Cell lines”**, change from five cell lines to four cell lines	In Materials and Methods section, the sentence “DMEM/F12 (Thermo Fisher Scientific, USA) containing 10% FBS (Gibico, NY, USA) was used to cultivate MAC-T, which was supplied by ATCC (USA). The cells were incubated in the humid incubator at 37°C and 5% CO_2_ during incubation.” is superfluous and should be deleted.
1223	Add the information of N-cadherin and E-cadherin antibodies in the section **“Materials and Methods-Western blot”**	Add the sentence “Since N-cadherin and E-cadherin have similar molecular weights, We used the 8% separation gel and selected two primary antibodies with a large distance between predicted locations, where the predicted position of N-cadherin (05-915, Sigma-Aldrich, 1:5000 dilution) was about 140kDa and the predicted position of E-cadherin (MAB3199Z, Sigma-Aldrich, 1:500 dilution) was about 100 kDa, in order to distinguish the two proteins as much as possible.” after the sentence “Membranes were then incubated with primary antibodies overnight at 4°C, using 5% skimmed milk.”.
1229	Replace the retracted reference Ref [26] in the section **“Reference”**	Replace Ref [26] with the new reference “Liu BX, Yang J, Zeng C, Chen Y. MACC1 Correlates with Tumor Progression and Immune Cell Infiltration of Colon Adenocarcinoma and is Regulated by the lncRNA ZFAS1/miR-642a-5p. Axis. J Oncol. 2022;2022:8179208. doi: 10.1155/2022/8179208. PMID: 36545127; PMCID: PMC9763013.”

The authors state that the scientific conclusions are unaffected. This correction was approved by the *Oncology Research* Editorial Office. The original publication has also been updated.

